# Storage stability of *Bacillus licheniformis* after Shijian-19 satellite flight

**DOI:** 10.3389/fmicb.2026.1875487

**Published:** 2026-08-19

**Authors:** Chunxing Li, Liu Lingying, Zhao Ren, Yujuan Li, Hua Liu

**Affiliations:** 1 Department of Pharmacy, Aerospace Center Hospital, Peking University Aerospace School of Clinical Medicine, Beijing, China; 2 School of Life Science, Beijing Institute of Technology, Beijing, China

**Keywords:** *Bacillus licheniformis*, biofilm formation, physiological activity, spaceflight, stability

## Abstract

**Objective:**

To evaluate the long-term stability of *Bacillus licheniformis live bacteria capsules (BL)* carried by the Shijian-19 satellite (SJ19) underground storage at −80 °C.

**Methods:**

*BL* was randomly divided into three groups: a spaceflight group (FS) exposed to 13.5-day orbital flight aboard SJ19 in single-dose packaging, a matched-packaging ground control (GS1), and an original commercial ground control (GS2). Bacterial growth curves, logarithmic-phase colony counts (LCC), acid–base tolerance, biofilm formation, adhesion ability, and amylase activity were measured immediately after recovery and after storage at −80 °C for 6 and 12 months. Treatment group and storage time effects were analyzed by two-way ANOVA or the Scheirer–Ray–Hare test, as appropriate.

**Results:**

All groups exhibited similar overall growth trends during storage. FS showed. comparable maximum cell densities (MCD) to GS1 throughout the experiment. MCD decreased significantly in all groups at month 6 (*p* < 0.001) and recovered to near baseline levels by month 12. FS had a significantly longer lag phase than GS1 at 0 and 6 months (*p* < 0.0001), while all groups showed pronounced lag-phase shortening at 12 months (*p* < 0.0001), and the intergroup difference gradually disappeared. The maximum specific growth rate declined over time, with no significant difference between FS and GS1. LCC in FS remained consistently lower than in GS1, reaching significance at months 6 (*p* = 0.01) and 12 (*p* = 0.0003). LCC increased over storage in all groups. Regarding enzyme activities, FS showed consistently lower *α*-amylase (*α*-AL) activity than GS1 at all time points (*p* < 0.01), and *α*-AL activity was significantly reduced in all groups at 6 and 12 months. FS also exhibited lower *β*-AL activity than GS1 at 6 and 12 months (*p* < 0.0001). Spaceflight significantly and persistently enhanced biofilm formation and adhesion ability relative to baseline. FS showed significantly higher biofilm formation (*p* < 0.01) and adhesion ability (*p* < 0.0001) than GS1 at months 0 and 6, with no significant difference in biofilm formation at month 12. Biofilm formation increased at month 6 (*p* < 0.0001) and decreased at month 12 (*p* ≤ 0.0001) in both FS and GS1. Adhesion ability decreased at month 6 (*p* < 0.0001) and recovered at month 12 (*p* < 0.0001) in all groups.

**Conclusion:**

Spaceflight is associated with remodeled growth metabolism and surface properties of *BL*, inducing growth retardation, decreased LCC, and inhibited *α*-AL activity, while enhancing biofilm formation and adhesion ability. During the 12-month storage, space-exposed strain exhibited time-dependent growth recovery, continuous *α*-AL reduction, and upregulation of biofilm/adhesion. This provides experimental evidence for evaluating space-stressed probiotics after orbital exposure and ground recovery.

## 1 Introduction

With the advancement of human space exploration, the effects of the space environment on biological systems have become a major research focus in space medicine and space biology (Wang et al., 2024). The space environment is characterized by unique conditions including microgravity, high-energy radiation, and extreme temperature fluctuations. These factors exert complex and profound impacts on astronauts’ physiological functions, leading to a range of health risks such as intestinal dysbiosis, bone loss, muscle atrophy, and impaired immune function (Bedree et al., 2023; Zhang et al., 2023). Meanwhile, they also profoundly alter the biological characteristics of microorganisms, thereby threatening astronaut health, spacecraft material integrity, and the stable operation of life-support systems in space (Nickerson et al., 2024).

Safe and effective medications are critical to protecting the physical and mental health of astronauts (Graebe et al., 2004). However, long-term space pharmacotherapy faces persistent challenges related to drug efficacy. Beyond the altered physiological status of astronauts, the poor stability of drugs under harsh spaceflight conditions represents a key contributing factor. Drug stability standards established under terrestrial conditions cannot accommodate the specificity of the space environment, making it difficult to evaluate drug stability during short- or long-term space missions. Unique space stressors—including microgravity, intense vibration, high vacuum, and strong radiation—may further trigger drug instability and exacerbate efficacy-related problems (Mehta and Bhayani, 2017).

*Bacillus licheniformis (BL)*, a prominent probiotic candidate, has garnered significant clinical attention for its therapeutic efficacy in ameliorating gastrointestinal dysbiosis, including diarrhea, abdominal distension, and dyspepsia (Ramirez-Olea et al., 2022; Zhou et al., 2023). Furthermore, it holds promise for mitigating spaceflight-associated health challenges, such as gut dysbiosis and compromised immune function in astronauts. Its probiotic mechanisms involve modulating gut microbiota equilibrium, inhibiting pathogen proliferation, and fortifying intestinal barrier integrity, while its immunomodulatory effects are mediated through macrophage activation and cytokine secretion (Pan et al., 2022; Wang et al., 2025). However, the stability of *BL* during and after spaceflight is critical for the development and application of space-grade probiotic formulations. Thus, conducting spaceflight-based studies is of paramount importance to ensure the long-term health of astronauts in orbit.

Current studies demonstrate that the space environment induces morphological changes, aberrant gene expression, and altered drug resistance and virulence factor expression in diverse bacterial species (Checinska Sielaff et al., 2019; Zea et al., 2017; Zhang et al., 2019). Phenotypic alterations include enlarged cell morphology, thickened cell walls, increased extracellular polysaccharide secretion, and modified biofilm formation (Zhao et al., 2019). Molecular changes involve reduced genome stability, elevated mutation rates, and altered expression of proteins related to energy metabolism, substance transport, and stress responses (Flores et al., 2023; Nickerson et al., 2000). Some bacteria even exhibit enhanced virulence, posing potential risks to space medical safety (Ding et al., 2020). However, systematic data on the biological changes of *BL* after spaceflight and its long-term stability following return to Earth remain lacking. Research into its stability is critical for ensuring scientific rigor and protecting astronaut health. Clarifying whether space-induced phenotypic changes are stably inherited is essential for understanding microbial environmental adaptation and assessing potential health hazards.

Shijian-19 (SJ19) is a key advanced technology experiment satellite under China’s 14th Five-Year Plan and provides a high-performance platform for high-microgravity space experiments. It was launched in September 2024, recovered in October 2024, and completed a 13.5-day in-orbit mission (CASC, 2024). In this study, viable *BL* capsules were loaded aboard SJ19. We systematically investigated the effects of spaceflight on the strain’s growth, physiological and biochemical characteristics, and tracked its stability during 1 year of storage at −80 °C after return to Earth. This study provides a basis for understanding the environmental adaptability of microorganisms and evaluating the potential health risks caused by changes in their characteristics, and lays a foundation for the research and development of space medical probiotic preparations.

## 2 Materials and methods

### 2.1 Materials

#### 2.1.1 Experimental samples

*BL* viable capsules (0.25 g per capsule, batch number: 202403122) produced by Northeast Pharmaceutical Group Shenyang No.1 Pharmaceutical Co., Ltd. were used in this study. These samples were carried by SJ19, launched on September 27, 2024, and returned on October 11, 2024, flying in a low Earth orbit of 330 kilometers for 13.5 days. Passive carrying was adopted with sealed containers, and the in-cabin conditions were natural temperature variation (approximately 0 to 35 °C), vacuum degree of about 1 × 10^−4^–1.3 × 10^−5^ Pa, microgravity of about 1 × 10^−7^ g, and radiation dose of 0.148 rad (Si) ~ 0.300 rad (Si).

Samples of *BL* from the same production batch were randomly divided into three groups. Due to satellite loading constraints, the flight group (FS) was packaged in single-dose sachets (10 capsules per pouch), and subjected to 13.5 days of in-orbit flight aboard SJ19. Ground control group 1 (GS1) received identical single-dose packaging (10 capsules per pouch) and was stored underground conditions (22 °C, 30–60% RH, normal gravity). Ground control group 2 (GS2) remained in unopened original packaging (36 capsules per bottle) and was stored under identical ground conditions as GS1.

At each time point (0, 6, and 12 months), fresh samples were taken from the original frozen stock, independently revived, and analyzed, yielding 3 independent packaging units per time point. For packaging effect analysis, the packaging container served as the experimental unit rather than individual capsules. All samples were stored protected from light in an ultra-low-temperature freezer at −80 °C after recovery to avoid repeated freezing and thawing.

#### 2.1.2 Main reagents and instruments

The Caco-2 cell line was purchased from the Chinese Academy of Sciences. Sodium chloride (NaCl), yeast extract, fluorescein isothiocyanate (FITC), *α*-amylase (*α*-AL) and *β*-amylase (*β*-AL) activity assay kit, 1% crystal violet stain, agarose, bovine serum albumin (BSA), and sterile PBS were purchased from Beijing Solarbio Science & Technology Co., Ltd. Tryptone was purchased from OXOID Ltd. (Basingstoke, UK). Sodium hydroxide (NaOH) and concentrated hydrochloric acid (HCl) were obtained from Beijing Chemical Plant.

Key instruments included a multifunctional microplate reader (Bio-Rad, USA), vortex mixer (Jinbeide Industry and Trade Co., Ltd.), constant-temperature shaker (Tianjin Test Instrument Co., Ltd.), constant-temperature incubator (Tianjin Test Instrument Co., Ltd.), autoclave (Binjiang Equipment Factory, China), water bath (Shanghai Jinghong Experimental Equipment Co., Ltd.), and −80 °C freezer (Thermo Fisher Scientific, USA).

### 2.2 Methods

#### 2.2.1 Strain activation and preservation

Lyophilized powder was extracted from capsules and inoculated into LB liquid medium at a ratio of 40 mL per capsule, followed by overnight incubation at 37 °C and 220 rpm. Overnight cultures were transferred to fresh LB medium at 3% inoculum size and cultured overnight at 37 °C and 220 rpm. Cultures were spread on LB solid medium and incubated at 37 °C for 24 h; single colonies were selected and purified by streak culture. Purified single colonies were inoculated into LB liquid medium and cultured to the logarithmic phase (OD₆₀₀ ≈ 0.6–0.8). Strains were preserved in 50% glycerol solution (bacterial suspension: glycerol = 1:1, final glycerol concentration 25%) and stored at −80 °C. All experiments were performed in three independent biological replicates (*n* = 3).

#### 2.2.2 Growth curve assay

Frozen strains were thawed in a 37 °C water bath and inoculated into LB medium at 5%. After incubation at 37 °C and 220 rpm, the optical density at 600 nm (OD₆₀₀) was measured every 2 h for 26 h to construct growth curves. Nonlinear regression of the growth data was performed using the Gompertz model to quantitatively evaluate the effects of spaceflight on the growth kinetics of *BL* cultures. The resulting model was used to estimate key kinetic parameters for each group, namely maximum cell density (MCD), lag phase duration, and maximum specific growth rate (μ_max_). All experiments were performed in three independent biological replicates (*n* = 3).

#### 2.2.3 Logarithmic-phase colony counting

Logarithmic-phase cultures were serially diluted to 10^−1^, 10^−2^, 10^−3^, 10^−4^, and 10^−5^. A 100 μL aliquot of the 10^−5^ dilution was spread on LB agar plates. After complete absorption, plates were incubated inverted at 37 °C overnight. Colony-forming units (CFU) were counted to calculate bacterial concentration. All experiments were performed in three independent biological replicates (*n* = 3).

#### 2.2.4 Acid–base tolerance assay

LB medium was adjusted to pH 5, 7, 9, 11, and 13. Strains were inoculated at 3% and cultured at 37 °C and 220 rpm for 24 h. Growth ability was evaluated by measuring OD₆₀₀. All experiments were performed in three independent biological replicates (*n* = 3).

#### 2.2.5 Biofilm formation assay

Biofilm formation was determined using crystal violet staining. Strains were inoculated into LB medium at 3% and cultured overnight at 37 °C and 220 rpm. Then, 10 μL of overnight culture was added to each well of a 96-well plate with 100 μL LB medium, followed by static incubation at 37 °C for 36 h. After washing with PBS, fixation with methanol, and staining with 1% crystal violet, biofilms were dissolved in 33% glacial acetic acid, and absorbance at 590 nm was measured. All experiments were performed in three independent biological replicates (*n* = 3).

#### 2.2.6 Adhesion ability assay

Strains were inoculated into LB medium at a volume ratio of 3% and cultured to logarithmic phase at 37 °C with shaking at 220 rpm. Bacterial cultures were centrifuged at 3,000 rpm for 10 min, and the cell pellets were washed twice with sterile PBS. Bacterial suspensions were diluted to 5 × 10^8^ CFU/mL using carbonate buffer, followed by staining with FITC at a final concentration 0.5 mg/mL. The staining reaction was performed at 37 °C for 1.5 h in the dark. After two additional washes with PBS, fluorescently labeled bacterial suspensions were prepared for subsequent use.

Caco-2 cells were seeded in 6-well plates at a density of 2 × 105 cells per well and cultured for 48 h to reach 90–95% confluence (fully formed intact cell monolayers). Subsequently, 1 mL of FITC-labeled bacterial suspension (1 × 10^8^ CFU/mL) was added to each well, resulting in a bacterium-to-Caco-2 cell ratio of approximately 500:1. The co-culture was incubated at 37 °C under 5% CO₂ for 1.5 h. Unbound bacteria were thoroughly removed by washing with PBS. The adherent bacteria together with Caco-2 cells were digested with 0.5 mL trypsin solution for 5 min, and the digestion was terminated by adding 0.5 mL complete medium.

The relative fluorescence intensity of each well was detected using a microplate reader, with excitation and emission wavelengths set at 485 nm and 530 nm, respectively. The initial number of Caco-2 cells in each well was identical, and no significant difference in cell number was observed among all groups after co-incubation, as verified by microscopic counting. Therefore, the raw fluorescence intensity was used to directly evaluate bacterial adhesion capacity without additional normalization. All experiments were performed in three independent biological replicates (*n* = 3).

#### 2.2.7 Enzyme activity assay

Amylase activity was measured using the 3,5-dinitrosalicylic acid (DNS) method, based on the ability of *α*-AL and *β*-AL to hydrolyze starch and produce reducing sugars. Bacterial cells were harvested by centrifugation at 3,000 rpm for 5 min. Then, 0.1 g of bacterial pellet was homogenized in 0.8 mL distilled water and incubated at room temperature for 15 min for enzyme extraction. After centrifugation at 6,000 × g for 10 min, the supernatant was adjusted to 10 mL as crude enzyme solution and further diluted 5-fold for testing. Taking advantage of the distinct biochemical properties of the two enzymes—*α*-AL is acid-labile while *β*-AL is thermolabile—we selectively inactivated one enzyme to quantify the activity of the other. All assays were performed in accordance with the manufacturer’s protocols. All experiments were performed in three independent biological replicates (*n* = 3).

#### 2.2.8 Storage stability assay

Strains were stored at −80 °C for 6 and 12 months. All above indices were re-detected to evaluate long-term stability after return to Earth.

### 2.3 Statistical analysis

All data were organized in Microsoft Office Excel 2016, and statistical analyses were performed using GraphPad Prism 9.0 (GraphPad Software, San Diego, CA, USA) and Stata 15.1 (Stata Corp LLC, College Station, TX, USA). The Shapiro–Wilk test was applied to verify data normality, and the Spearman rank correlation test was applied to assess homogeneity of variance. Normally distributed data are expressed as mean ± standard deviation (mean ± SD). Data violating normality or homogeneity of variance were log-transformed. Two-way ANOVA was used to examine the main effects of treatment group and storage time, along with their interaction effect when the transformed data met the statistical assumptions. Simple-effects analysis was conducted if a significant interaction was detected. Tukey’s *post-hoc* multiple comparisons were used for pairwise comparisons within and between groups at each time point. Additionally, Dunnett’s test was performed to compare the FS and GS2 groups against the GS1 control group.

For data that still failed to satisfy normality and homogeneity of variance after log-transformation, nonparametric analyses were performed. The Scheirer–Ray–Hare test was used to evaluate the main and interactive effects of treatment and storage time. Significant interactions were further analyzed via the Kruskal–Wallis test for simple effects. Pairwise comparisons were conducted using the Mann–Whitney *U* test with Bonferroni correction. A *p*-value < 0.05 was defined as statistically significant. Significance levels: ns, not significant (*p* ≥ 0.05); *p < 0.05, **p < 0.01, ***p < 0.001, ****p < 0.0001.

## 3 Results

### 3.1 Growth curves and growth kinetic parameters

**Figure 1:**
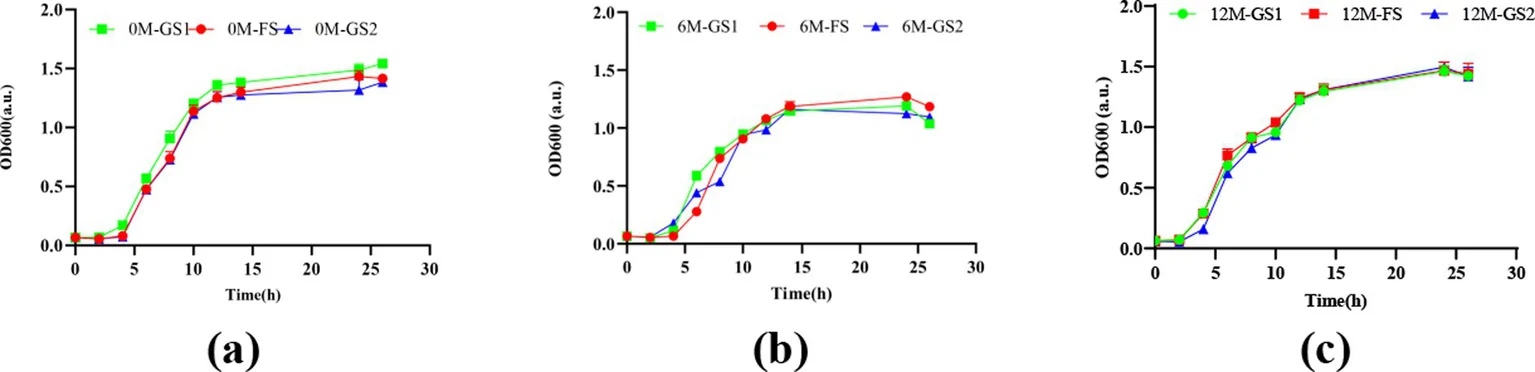
Determination of strain growth curve **(a)** Growth curve at 0 month after retrieval; **(b)** growth curve at 6 months after retrieval; **(c)** growth curve at 12 months after retrieval.

**Figure 2:**
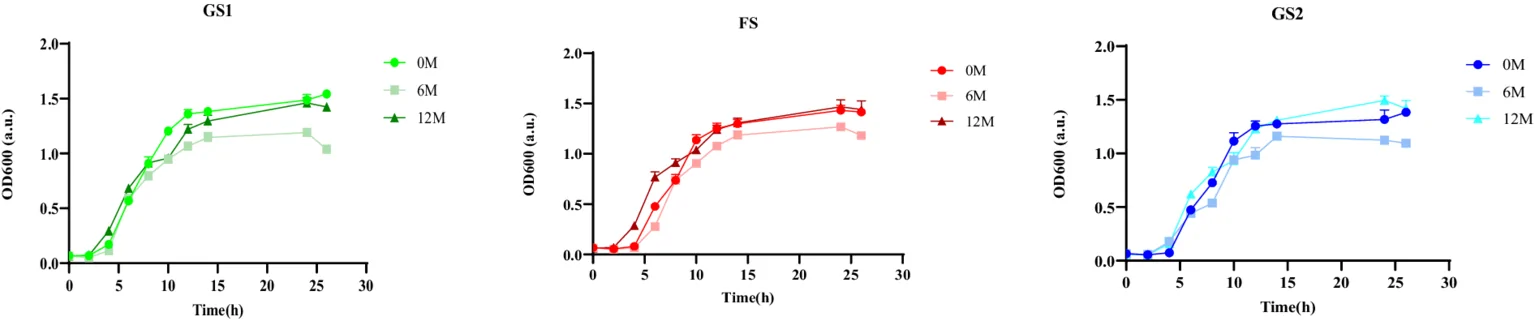
Comparison of growth curves of strains in each group before and after 12 months of storage.

The three groups exhibited similar overall growth trends at 0, 6 and 12 months of storage (Figures 1, 2). At all three time points, *BL* strains from each group displayed typical bacterial growth curves (Figures 1, 2).

Two-way ANOVA revealed a significant interaction between treatment group and storage time (*p* < 0.05) (Table 1). Simple main effect analysis indicated that both the lag phase and μ_max_ differed substantially across groups at each time point, while all tested kinetic parameters varied significantly over storage time within individual groups (*p* < 0.0001).

**Table 1:** Effects of treatment group and storage duration on key parameters in *Bacillus licheniformis*.

Parameters	Treatment group (TG)			Storage duration (SD)			TG × SD interaction		
	Total var. (%)	*F*	*p*	Total var. (%)	*F*	*p*	Total var. (%)	*F*	*p*
Maximum cell density	2.06	1.97	0.17	82.09	78.67	<0.0001	6.47	3.10	0.04
Lag time	8.89	83.23	<0.0001	73.56	688.90	<0.0001	16.59	77.69	<0.0001
Maximum specific growth rate	6.80	7.43	0.004	62.41	68.15	<0.0001	22.55	12.31	<0.0001
Logarithmic-phase colony counts	37.97	26.27	<0.0001	40.02	27.69	<0.0001	15.51	5.37	0.02
Acid–base tolerance (pH 7)	2.32	1.84	0.19	66.81	52.89	<0.0001	19.50	7.72	0.001
Acid–base tolerance (pH 9)	14.16	19.80	<0.0001	69.85	97.70	<0.0001	9.56	6.68	0.002
Biofilm formation ability	16.23	97.83	<0.0001	48.51	292.40	<0.0001	33.77	101.80	<0.0001
Adhesion capacity	49.29	1264.00	<0.0001	49.48	1269.00	<0.0001	0.87	11.20	<0.0001
Total *α*-amylase	2.42	499.60	<0.0001	96.46	19,911	<0.0001	1.08	111.30	<0.0001
Total *β*-amylase	38.64	132.70	<0.0001	16.60	57.02	<0.0001	42.14	72.35	<0.0001
Intracellular *α*-amylase	1.95	695.90	<0.0001	95.35	34115.00	<0.0001	2.68	479.00	<0.0001
Extracellular *α*-amylase	25.33	233.00	<0.0001	65.09	598.60	<0.0001	8.60	39.54	<0.0001
Intracellular *β*-amylase	57.27	40.89	<0.0001	16.63	11.87	0.0005	13.49	4.82	0.01
Extracellular *β*-amylase	8.78	50.59	<0.0001	39.58	228.10	<0.0001	50.08	144.30	<0.0001

Data show the percentage of total variation, *F*-value, and *p*-value. Two-way ANOVA was used for data with normal distribution and homogeneous variance; the Scheirer–Ray–Hare test was applied for non-normally distributed data. *n* = 3 biological replicates.

**Figure 3:**
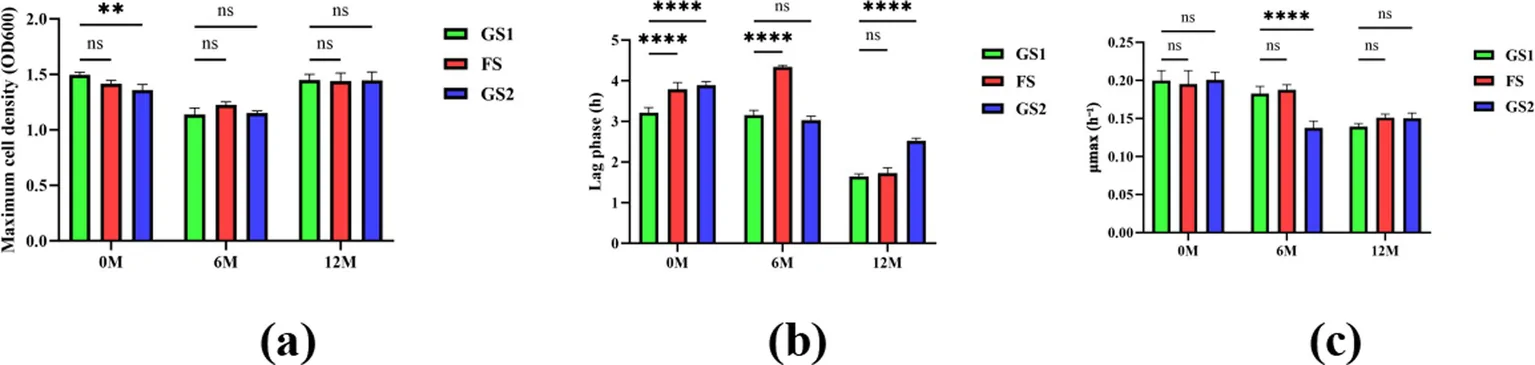
Dynamics of growth kinetic parameters in different groups during storage **(a)** Maximum cell density; **(b)** lag phase; **(c)** maximum specific growth rate (*μ*_max_). ns, not significant (p ≥ 0.05); **p < 0.01,****p < 0.0001.

Dunnett’s test (Table 2) revealed that at month 0, FS had a longer lag phase (*p* < 0.0001) but similar MCD and μ_max_ relative to GS1. GS2 presented lower MCD and longer lag phase (both *p* < 0.01), with no significant change in μ_max_. At month 6, only the lag phase of FS and μ_max_ of GS2 differed significantly from GS1 (both *p* < 0.0001), and MCD was comparable among groups. At month 12, FS showed no differences from GS1 in most parameters. GS2 had a distinctly shorter lag phase (*p* < 0.0001), with no other significant differences observed among groups (Figure 3).

**Table 2:** Temporal changes key parameters of *Bacillus licheniformis* under different storage durations.

Parameters	Group	At 0 month	At 6 month	At 12 month	Adjusted *p-*value		
					0 M vs 6 M	0 M vs 12 M	6 M vs 12 M
Maximum cell density	GS1	1.50 ± 0.02	1.14 ± 0.06	1.45 ± 0.05	<0.0001	0.48	<0.0001
	FS	1.41 ± 0.03	1.23 ± 0.03	1.44 ± 0.08	0.0008	0.84	0.0002
	GS2	1.36 ± 0.05	1.15 ± 0.02	1.44 ± 0.08	0.0003	0.12	<0.0001
Lag time	GS1	3.22 ± 0.13	3.16 ± 0.12	1.64 ± 0.06	0.77	<0.0001	<0.0001
	FS	3.79 ± 0.17	4.35 ± 0.04	1.73 ± 0.13	<0.0001	<0.0001	<0.0001
	GS2	3.89 ± 0.09	3.03 ± 0.10	2.52 ± 0.06	<0.0001	<0.0001	<0.0001
Maximum specific growth rate	GS1	0.20 ± 0.01	0.18 ± 0.01	0.14 ± 0.00	0.14	<0.0001	<0.0001
	FS	0.20 ± 0.02	0.19 ± 0.01	0.15 ± 0.00	0.71	<0.0001	0.0004
	GS2	0.20 ± 0.01	0.14 ± 0.01	0.15 ± 0.01	<0.0001	<0.0001	0.15
Logarithmic-phase colony counts	GS1	5.70 × 10^7^ ± 4.24 × 10^6^	1.37 × 10^8^ ± 7.07 × 10^5^	2.12 × 10^8^ ± 2.76 × 10^7^	0.01	<0.0001	0.01
	FS	2.10 × 10^7^ ± 5.66 × 10^6^	6.70 × 10^7^ ± 1.41 × 10^7^	9.10 × 10^7^ ± 5.66 × 10^6^	0.10	0.02	0.47
	GS2	1.11 × 10^8^ ± 2.12 × 10^7^	1.28 × 10^8^ ± 2.83 × 10^6^	1.40 × 10^8^ ± 4.45 × 10^7^	0.68	0.36	0.83
Acid–base tolerance (pH 7)	GS1	0.82 ± 0.03	0.67 ± 0.01	0.68 ± 0.02	0.15	0.14	1.00
	FS	0.83 ± 0.06	0.60 ± 0.00	0.68 ± 0.01	0.15	0.15	0.15
	GS2	0.82 ± 0.04	0.69 ± 0.02	0.65 ± 0.01	0.15	0.15	0.15
Acid–base tolerance (pH 9)	GS1	0.87 ± 0.05	0.60 ± 0.00	0.71 ± 0.03	0.15	0.15	0.15
	FS	0.87 ± 0.05	0.61 ± 0.01	0.71 ± 0.01	0.15	0.15	0.15
	GS2	0.91 ± 0.03	0.75 ± 0.02	0.74 ± 0.02	0.15	0.15	0.55
Biofilm formation ability	GS1	0.63 ± 0.11	2.79 ± 0.11	2.09 ± 0.16	<0.0001	<0.0001	0.0001
	FS	1.14 ± 0.13	3.71 ± 0.14	2.34 ± 0.04	<0.0001	<0.0001	<0.0001
	GS2	0.66 ± 0.24	0.70 ± 0.28	2.68 ± 0.07	0.95	<0.0001	<0.0001
Adhesion capacity	GS1	1,103 ± 24.58	955.30 ± 4.04	1,446 ± 52.56	<0.0001	<0.0001	<0.0001
	FS	1,665 ± 13.23	1,430 ± 8.19	1,984 ± 26.39	<0.0001	<0.0001	<0.0001
	GS2	1,499 ± 12.06	1,237 ± 14.98	1,901 ± 21.08	<0.0001	<0.0001	<0.0001
Total *α*-amylase	GS1	0.37 ± 0.00	0.03 ± 0.01	0.03 ± 0.00	<0.0001	<0.0001	0.58
	FS	0.28 ± 0.00	0.01 ± 0.00	0.02 ± 0.00	<0.0001	<0.0001	0.0003
	GS2	0.37 ± 0.00	0.06 ± 0.01	0.05 ± 0.00	<0.0001	<0.0001	0.0002
Total *β*-amylase	GS1	1.29 ± 0.01	3.33 ± 0.10	1.54 ± 0.09	<0.0001	0.10	<0.0001
	FS	1.07 ± 0.01	1.16 ± 0.23	0.71 ± 0.26	0.72	0.01	0.002
	GS2	1.45 ± 0.01	1.18 ± 0.20	1.60 ± 0.06	0.08	0.42	0.005
Intracellular *α*-amylase	GS1	0.33 ± 0.00	0.01 ± 0.00	0.03 ± 0.00	<0.0001	<0.0001	<0.0001
	FS	0.22 ± 0.00	0.00 ± 0.00	0.02 ± 0.00	<0.0001	<0.0001	<0.0001
	GS2	0.27 ± 0.00	0.03 ± 0.00	0.02 ± 0.00	<0.0001	<0.0001	0.002
Extracellular *α*-amylase	GS1	0.04 ± 0.00	0.02 ± 0.01	0.00 ± 0.00	<0.0001	<0.0001	0.003
	FS	0.06 ± 0.00	0.01 ± 0.00	0.00 ± 0.00	<0.0001	<0.0001	0.62
	GS2	0.10 ± 0.00	0.03 ± 0.00	0.02 ± 0.00	<0.0001	<0.0001	0.04
Intracellular *β*-amylase	GS1	1.18 ± 0.00	1.01 ± 0.03	1.17 ± 0.11	>0.05	>0.05	>0.05
	FS	0.95 ± 0.00	0.40 ± 0.08	0.46 ± 0.28	>0.05	>0.05	>0.05
	GS2	1.07 ± 0.01	1.00 ± 0.20	1.38 ± 0.07	>0.05	>0.05	>0.05
Extracellular *β*-amylase	GS1	0.11 ± 0.01	2.32 ± 0.12	0.38 ± 0.03	<0.0001	<0.0001	<0.0001
	FS	0.12 ± 0.01	0.76 ± 0.21	0.24 ± 0.03	<0.0001	<0.0001	<0.0001
	GS2	0.38 ± 0.00	0.18 ± 0.03	0.22 ± 0.02	<0.0001	0.0003	0.28

Data are presented as mean ± standard deviation (SD), *n* = 3, biological replicates. 0 M: at 0 month, 6 M: at 6 month, 12 M: at 12 month.

**Figure 4:**
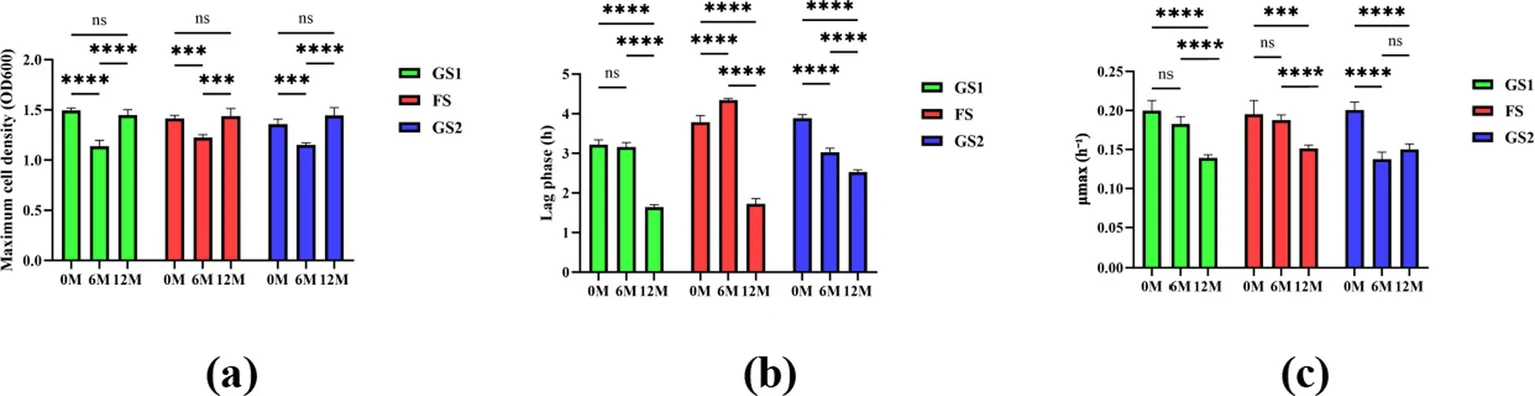
Dynamics of growth kinetic parameters across storage time in each group **(a)** Maximum cell density; **(b)** lag phase; **(c)** maximum specific growth rate (*μ*_max_). ns, not significant (*p* ≥ 0.05); ***p < 0.001,****p < 0.0001.

Intragroup Tukey’s *post-hoc* comparisons revealed temporal dynamics of growth parameters during storage (Table 3). MCD dropped significantly at month 6 in all groups (all adjusted *p* < 0.001), then rose sharply at month 12 (all *p* < 0.0001) and returned to the baseline level (Figure 4). The lag phase of GS1 remained stable from month 0 to 6 (*p* = 0.77). During the same period, the lag phase increased significantly in FS but decreased markedly in GS2 (all *p* < 0.0001). All groups had much shorter lag phases at month 12 (all *p* < 0.0001). For μ_max_, GS1 and FS remained unchanged in the first 6 months and declined afterwards. In GS2, this parameter decreased rapidly from month 0 to 6 (*p* < 0.0001) and plateaued subsequently (*p* = 0.30) (Figure 4).

**Table 3:** The inter-group differences among *Bacillus licheniformis* in different treatment groups.

Parameters	Storage duration	Comparison	Adjusted *p-*value	Parameters	Storage duration	Comparison	Adjusted *p-*value
Maximum cell density	0 M	GS1 vs. FS	0.11	Biofilm formation ability	0 M	GS1 vs. FS	0.002
		GS1 vs. GS2	0.01			GS1 vs. GS2	0.96
	6 M	GS1 vs. FS	0.09		6 M	GS1 vs. FS	<0.0001
		GS1 vs. GS2	0.93			GS1 vs. GS2	<0.0001
	12 M	GS1 vs. FS	0.96		12 M	GS1 vs. FS	0.136
		GS1 vs. GS2	1.00			GS1 vs. GS2	0.0005
Lag time	0 M	GS1 vs. FS	<0.0001	Total *α*-amylase	0 M	GS1 vs. FS	<0.0001
		GS1 vs. GS2	<0.0001			GS1 vs. GS2	0.44
	6 M	GS1 vs. FS	<0.0001		6 M	GS1 vs. FS	<0.0001
		GS1 vs. GS2	0.26			GS1 vs. GS2	<0.0001
	12 M	GS1 vs. FS	0.53		12 M	GS1 vs. FS	0.02
		GS1 vs. GS2	<0.0001			GS1 vs. GS2	0.0003
Maximum specific growth rate	0 M	GS1 vs. FS	0.79	Total *β*-amylase	0 M	GS1 vs. FS	0.13
		GS1 vs. GS2	0.99			GS1 vs. GS2	0.32
	6 M	GS1 vs. FS	0.75		6 M	GS1 vs. FS	<0.0001
		GS1 vs. GS2	<0.0001			GS1 vs. GS2	<0.0001
	12 M	GS1 vs. FS	0.14		12 M	GS1 vs. FS	<0.0001
		GS1 vs. GS2	0.19			GS1 vs. GS2	0.86
Logarithmic-phase colony counts	0 M	GS1 vs. FS	0.17	Intracellular *α*-amylase	0 M	GS1 vs. FS	<0.0001
		GS1 vs. GS2	0.04			GS1 vs. GS2	<0.0001
	6 M	GS1 vs. FS	0.01		6 M	GS1 vs. FS	<0.0001
		GS1 vs. GS2	0.88			GS1 vs. GS2	<0.0001
	12 M	GS1 vs. FS	0.0003		12 M	GS1 vs. FS	0.002
		GS1 vs. GS2	0.01			GS1 vs. GS2	0.02
Acid–base tolerance (pH 7)	0 M	GS1 vs. FS	0.09	Extracellular *α*-amylase	0 M	GS1 vs. FS	<0.0001
		GS1 vs. GS2	0.09			GS1 vs. GS2	<0.0001
	6 M	GS1 vs. FS	0.10		6 M	GS1 vs. FS	0.01
		GS1 vs. GS2	0.25			GS1 vs. GS2	<0.0001
	12 M	GS1 vs. FS	1.00		12 M	GS1 vs. FS	0.90
		GS1 vs. GS2	0.24			GS1 vs. GS2	<0.0001
Acid–base tolerance (pH 9)	0 M	GS1 vs. FS	1.00	Intracellular *β*-amylase	0 M	GS1 vs. FS	0.09
		GS1 vs. GS2	1.00			GS1 vs. GS2	0.09
	6 M	GS1 vs. FS	0.10		6 M	GS1 vs. FS	0.10
		GS1 vs. GS2	0.10			GS1 vs. GS2	1.00
	12 M	GS1 vs. FS	1.00		12 M	GS1 vs. FS	0.10
		GS1 vs. GS2	0.25			GS1 vs. GS2	0.10
Adhesion capacity	0 M	GS1 vs. FS	<0.0001	Extracellular *β*-amylase	0 M	GS1 vs. FS	0.60
		GS1 vs. GS2	<0.0001			GS1 vs. GS2	<0.0001
	6 M	GS1 vs. FS	<0.0001		6 M	GS1 vs. FS	<0.0001
		GS1 vs. GS2	<0.0001			GS1 vs. GS2	<0.0001
	12 M	GS1 vs. FS	<0.0001		12 M	GS1 vs. FS	0.002
		GS1 vs. GS2	<0.0001			GS1 vs. GS2	0.0003

0 M: at 0 month, 6 M: at 6 month, 12 M: at 12 month. *n* = 3, biological replicates.

### 3.2 Logarithmic-phase colony counting

Two-way ANOVA indicated that treatment group, storage duration and their interaction all significantly affected LCC (*p* < 0.05), explaining 37.97, 40.02 and 15.51% of total variation, respectively (Table 1).

**Figure 5:**
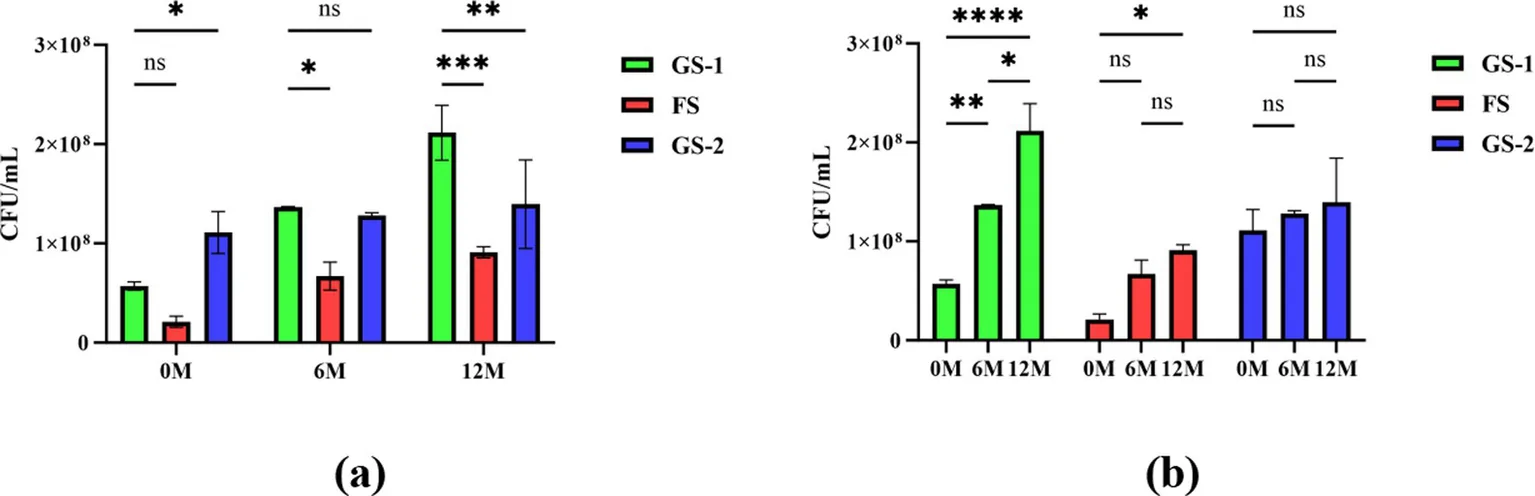
Effects of treatment and storage time on logarithmic-phase colony counts **(a)** Logarithmic-phase colony counts in different groups during storage, analyzed by Dunnett’s multiple comparisons test. **(b)** Logarithmic-phase colony counts across storage time within each group, analyzed by Tukey’s *post-hoc* test. ns, not significant (p ≥ 0.05); *p < 0.05, **p < 0.01, ***p < 0.001, ****p < 0.0001.

Dunnett’s test (Table 2) with GS1 as the reference showed no significant difference between GS1 and FS at month 0, while GS2 had a markedly higher LCC (*p* = 0.04). At month 6, FS differed significantly from GS1 (*p* = 0.01), whereas GS2 was comparable to the control. At month 12, both FS and GS2 exhibited distinct differences relative to GS1 (*p* = 0.0003 and *p* = 0.01, respectively) (Figure 5).

Intragroup Tukey’s *post-hoc* comparisons further illustrated temporal changes in LCC (Table 3). LCC of GS1 increased continuously throughout storage, with significant differences detected among all time points. In the FS group, LCC was significantly higher at month 12 than at month 0 (*p* = 0.02), while no other pairwise differences were observed. No obvious temporal variation was found in the GS2 group across the whole storage period (Figure 5).

### 3.3 Acid–base tolerance analysis

**Figure 6:**
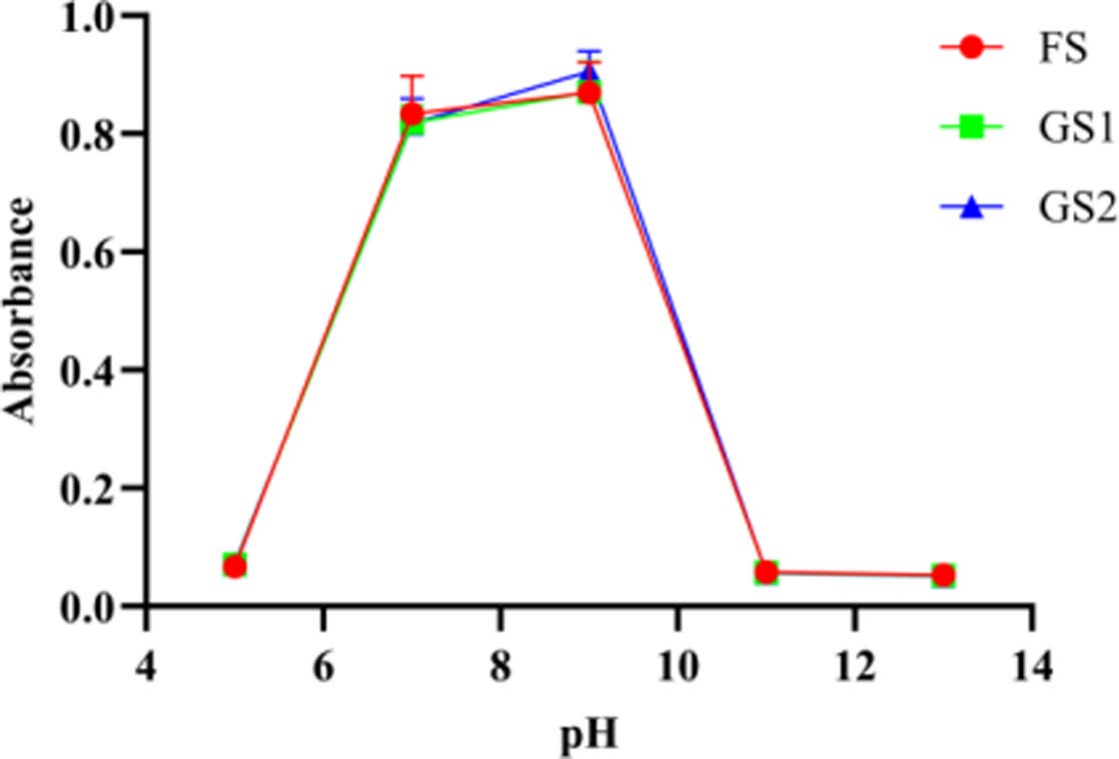
pH tolerance profiles of *BL* at month 0.

We assessed the acid–base tolerance of the strain at pH 5, 7, 9, 11 and 13 at month 0 (Figure 6). Only minor inter-group differences were observed at pH 7 and pH 9, while no obvious variations were detected at pH 5, 11 and 13. Based on these findings, subsequent survival assays at 6 and 12 months of storage were conducted only at pH 7 and pH 9. Therefore, we mainly present the data obtained under these two pH conditions in the following content.

Scheirer–Ray–Hare nonparametric analysis showed that storage duration exerted a strong effect on bacterial tolerance at pH 7 and pH 9 (Table 1). At pH 7, significant effects were observed for duration and the group-duration interaction (*p* ≤ 0.001), while group effect was non-significant (*p* = 0.19). All main and interaction effects were significant at pH 9 (*p* ≤ 0.01).

**Figure 7:**
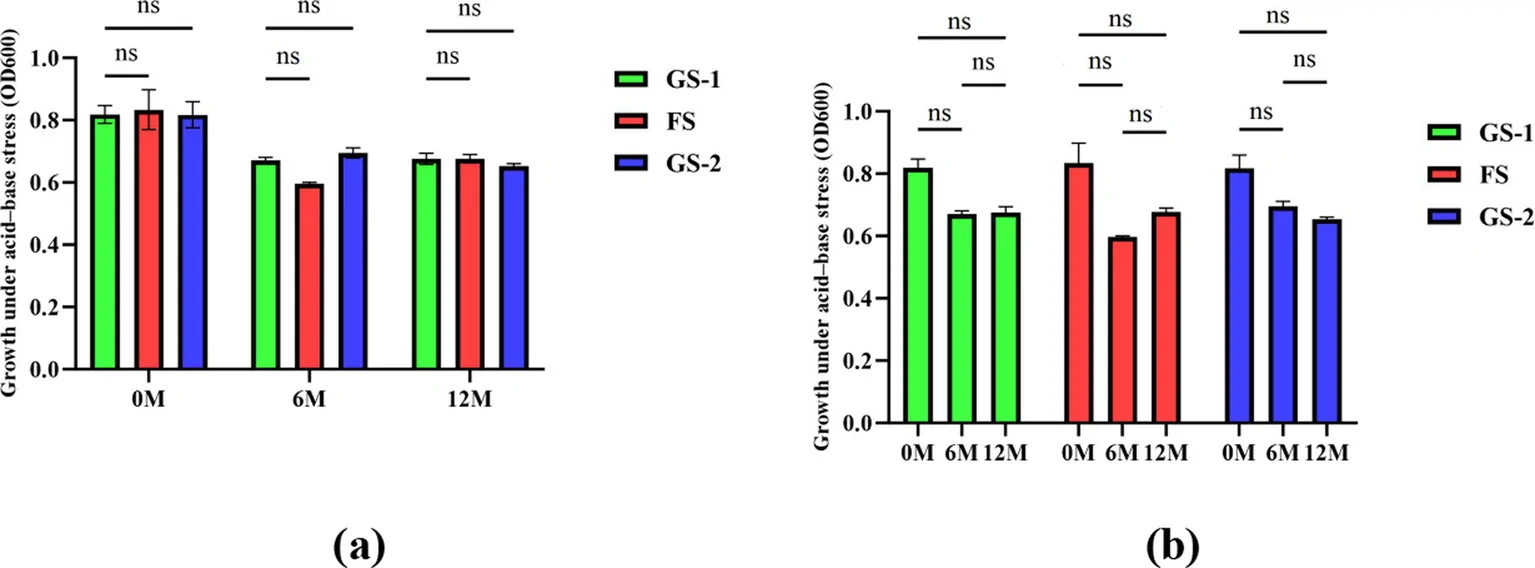
Effects of treatment and storage duration on acid–base tolerance at pH 7. **(a)** Acid–base tolerance in different groups during storage, analyzed by Mann–Whitney *U* test with Bonferroni correction. **(b)** Acid–base tolerance across storage time within each group, analyzed by Mann–Whitney *U* test with Bonferroni correction. ns, not significant.

**Figure 8:**
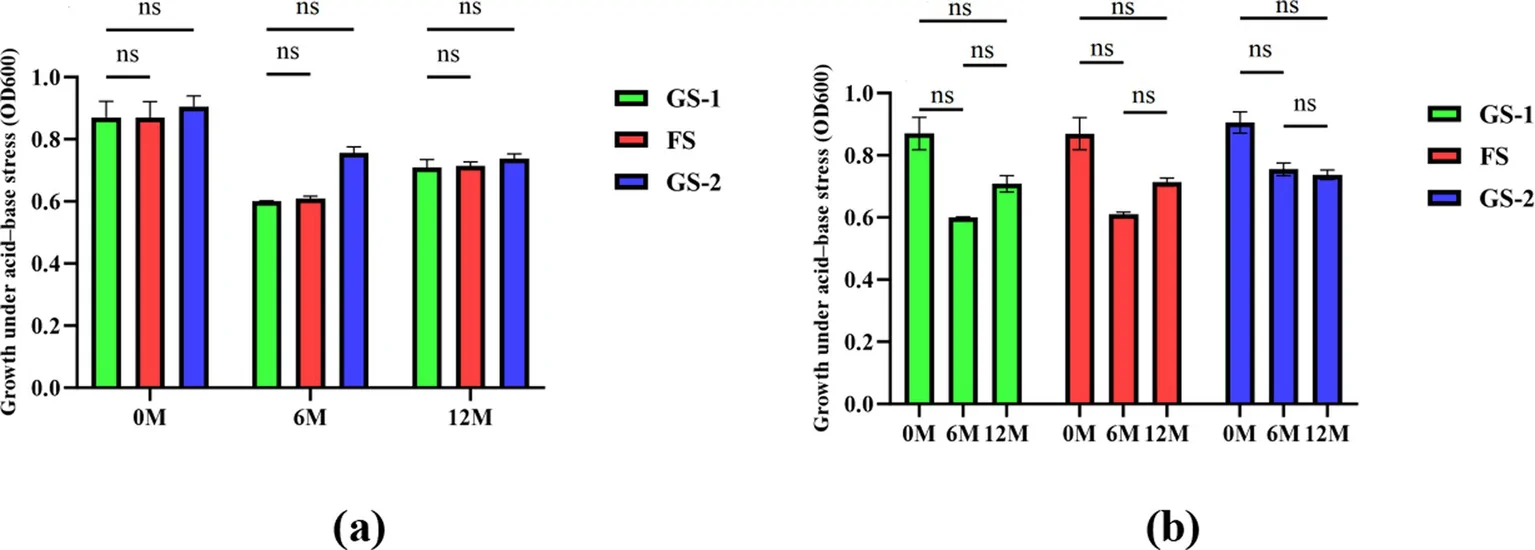
Effects of treatment and storage duration on acid–base tolerance at pH 9. **(a)** Acid–base tolerance in different groups during storage, analyzed by Mann–Whitney *U* test with Bonferroni correction. **(b)** Acid–base tolerance across storage time within each group, analyzed by Mann–Whitney *U* test with Bonferroni correction. ns, not significant (*p* ≥ 0.05).

Mann–Whitney *U* test with Bonferroni correction (Table 2) showed no significant intergroup differences in acid–base tolerance at either pH 7 or pH 9 across all time points, with GS1 as the control (all adjusted *p* > 0.05) (Figures 7, 8).

Mann–Whitney *U* test with Bonferroni correction (Table 2) showed no significant differences in acid–base tolerance at either pH 7 or pH 9 across all time points (0 M vs. 6 M, 0 M vs. 12 M, 6 M vs. 12 M) within the GS-1, FS, and GS-2 groups (all adjusted *p* > 0.05) (Figures 7, 8).

### 3.4 Biofilm formation assay

Two-way ANOVA confirmed that treatment group, storage duration and their interaction all significantly affected biofilm formation (*p* < 0.0001) (Table 1).

**Figure 9:**
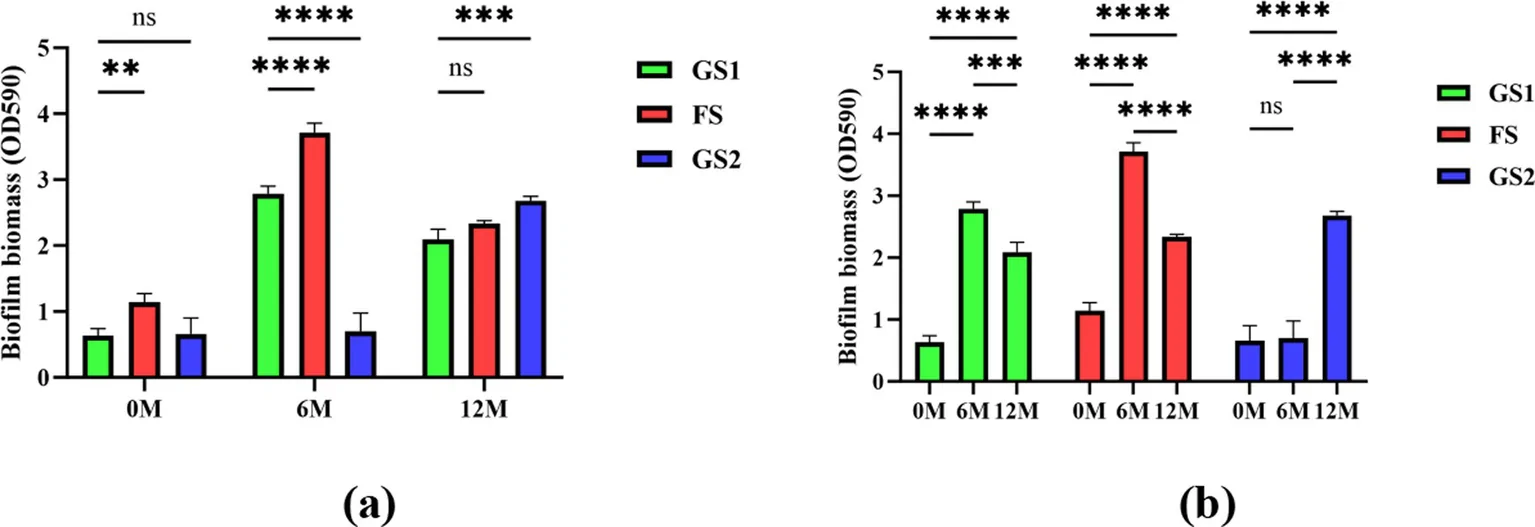
Effects of treatment and storage time on biofilm formation. **(a)** Biofilm formation in different groups during storage, analyzed by Dunnett’s multiple comparisons test. **(b)** Biofilm formation across storage time within each group, analyzed by Tukey’s *post-hoc* test. ns, not significant (*p* ≥ 0.05); **p < 0.01, ***p < 0.001, ****p < 0.0001.

Dunnett’s test (Table 2) showed that at month 0, FS exhibited significantly stronger biofilm formation than GS1 (*p* = 0.002), while no difference was observed between GS2 and GS1. At month 6, both FS and GS2 differed markedly from GS1 (both *p* < 0.0001). At month 12, only GS2 showed a significant difference relative to GS1 (*p* = 0.0005), and FS was comparable to the control group (Figure 9).

Tukey’s *post-hoc* test was used to analyze temporal changes within each group (Table 3). For GS1 and FS, biofilm formation capacity increased sharply from month 0 to 6 and decreased significantly at month 12 (all *p* < 0.0001). In the GS2 group, biofilm formation remained stable over the first 6 months (*p* = 0.95), but rose dramatically at month 12 compared with the earlier two time points (all *p* < 0.0001) (Figure 9).

### 3.5 Adhesion ability assay

Two-way ANOVA showed significant main effects of treatment group and storage duration, as well as a significant interaction between the two factors on bacterial adhesion ability (all *p* < 0.0001). Treatment group, storage duration and their interaction contributed 49.29, 49.48 and 0.87% to the total variation, respectively (Table 1).

**Figure 10:**
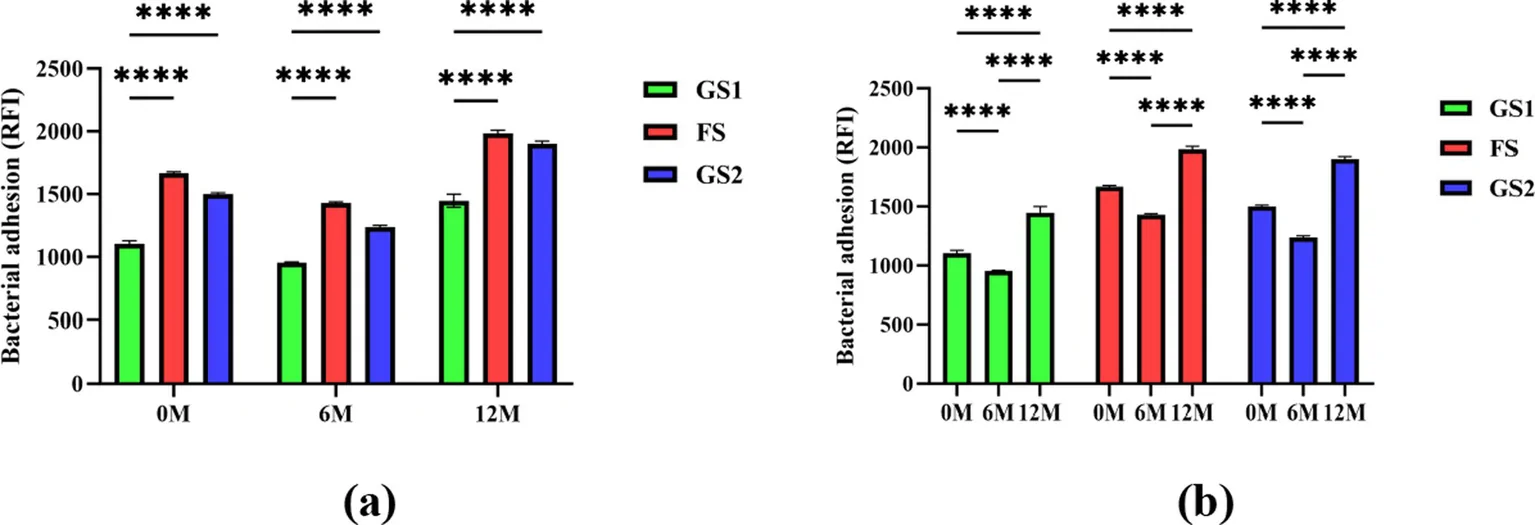
Effects of treatment and storage time on bacterial adhesion ability **(a)** Bacterial adhesion ability in different groups during storage, analyzed by Dunnett’s multiple comparisons test. **(b)** Bacterial adhesion ability across storage time within each group, analyzed by Tukey’s *post-hoc* test. ****p < 0.0001.

Dunnett’s test (Table 2) revealed that both FS and GS2 exhibited significantly higher adhesion ability than GS1 at month 0, 6 and 12 (all *p* < 0.0001) (Figure 10).

Tukey’s *post-hoc* intragroup comparisons (Table 3) indicated that adhesion capacity changed remarkably over storage time in all three groups. For each group, adhesion ability decreased significantly from month 0 to month 6, followed by a sharp increase at month 12; significant differences were detected between every two time points (all *p* < 0.0001) (Figure 10).

### 3.6 Amylase activity assay

The total activity of intracellular and extracellular *α*-AL and *β*-AL was significantly affected by treatment group, storage duration, and their interaction (all *p* < 0.0001) (Table 1).

**Figure 11:**
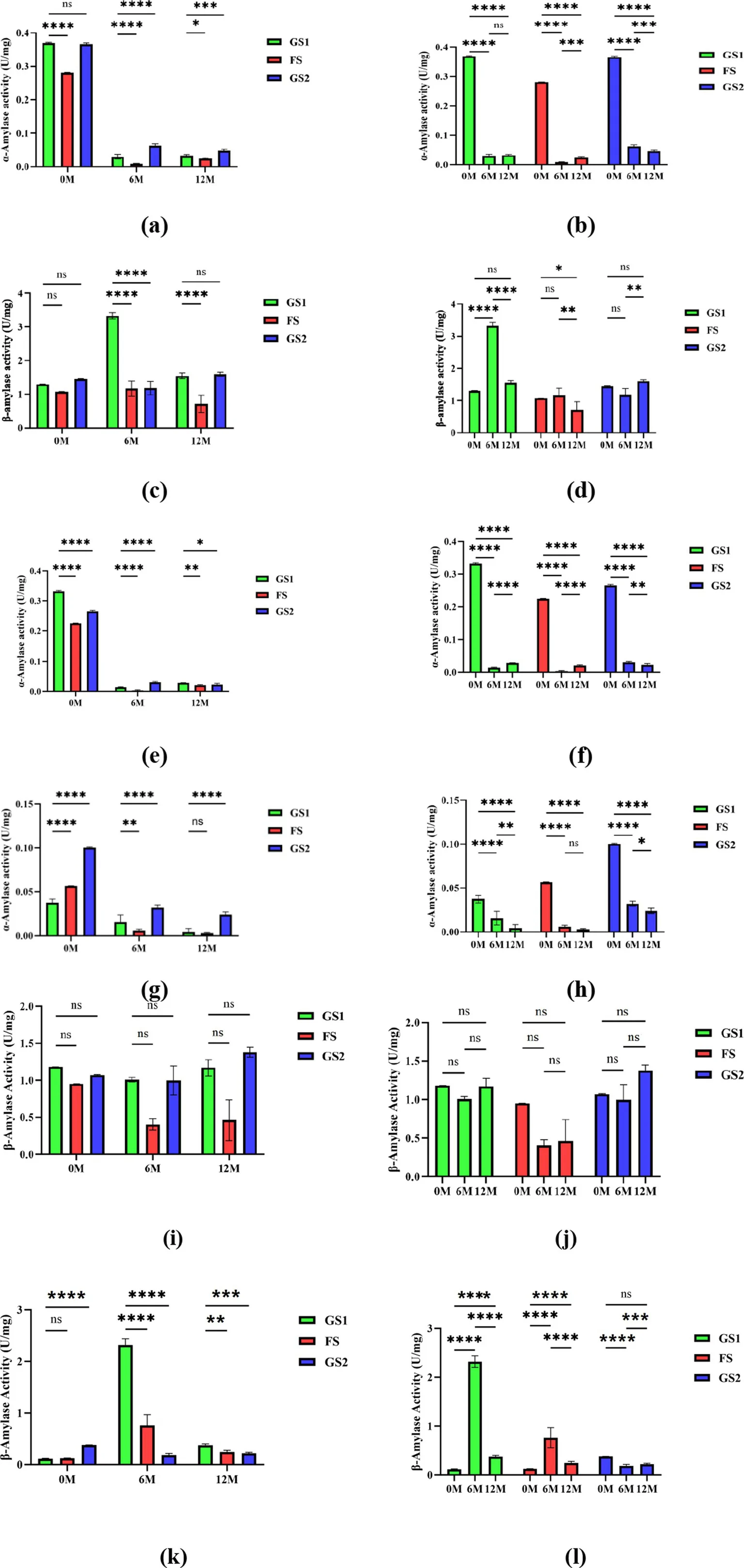
Effects of treatment and storage time on intracellular and extracellular amylase activities: **(a)** Total *α*-amylase activity in different groups, analyzed by Dunnett’s multiple comparisons test; **(b)** total *α*-amylase activity across storage time, analyzed by Tukey’s *post-hoc* test; **(c)** total *β*-amylase activity in different groups, analyzed by Dunnett’s multiple comparisons test; **(d)** total *β*-amylase activity across storage time, analyzed by Tukey’s *post-hoc* test; **(e)** intracellular *α*-amylase activity in different groups, analyzed by Dunnett’s multiple comparisons test; **(f)** intracellular *α*-amylase activity across storage time, analyzed by Tukey’s *post-hoc* test; **(g)** extracellular *α*-amylase activity in different groups, analyzed by Dunnett’s multiple comparisons test; **(h)** extracellular *α*-amylase activity across storage time, analyzed by Tukey’s *post-hoc* test; **(i)** intracellular *β*-amylase activity in different groups, analyzed by Mann–Whitney *U* test with Bonferroni correction; **(j)** intracellular *β*-amylase activity across storage time, analyzed by Mann–Whitney *U* test with Bonferroni correction; **(k)** extracellular *β*-amylase activity in different groups, analyzed by Dunnett’s multiple comparisons test; **(l)** extracellular *β*-amylase activity across storage time, analyzed by Tukey’s *post-hoc* test. ns, not significant (*p* ≥ 0.05); *p < 0.05, **p < 0.01, ***p < 0.001, ****p < 0.0001.

Dunnett’s test (Table 2) showed total *α*-AL activity in GS1 was significantly higher than that in FS at all time points (*p* < 0.0001 at 0 M and 6 M, *p* = 0.02 at 12 M). There was no significant difference between GS1 and GS2 at 0 M (*p* = 0.44), while GS1 presented significantly higher activity at 6 M (*p* < 0.0001) and 12 M (*p* = 0.0003) (Figure 11a). At month 0, total *β*-AL activity in FS was comparable to that in GS1. At months 6 and 12, it was significantly lower in FS than in GS1 (Figure 11c).

Tukey’s test indicated that total *α*-AL activity decreased markedly from 0 M to 6 M and 12 M across all groups (all *p* < 0.0001). No significant difference was observed between 6 M and 12 M in the GS1 group (*p* = 0.58). By contrast, the FS and GS2 groups exhibited significant differences between 6 M and 12 M (*p* = 0.0003 and *p* = 0.0002, respectively) (Figure 11b). In the FS group, total *β*-AL activity was similar at month 6 and month 0, but significantly decreased at month 12 relative to both. In the GS1 group, activity was significantly elevated at month 6 compared with month 0, then reduced at month 12 to a level comparable to month 0 (Figure 11d).

Furthermore, two-way ANOVA was performed on extracellular and intracellular *α*-AL activity. For *β*-AL activity, log-transformed extracellular data were analyzed by two-way ANOVA, whereas intracellular data were analyzed using the Scheirer–Ray–Hare test. Both analyses revealed that treatment group, storage time, and their interaction all significantly influenced both intracellular and extracellular *α*-AL and *β*-AL activities (all *p* < 0.01) (Table 1). Among these factors, storage time was the primary determinant affecting *α*-AL activity. Results of pairwise comparisons are presented in Tables 2, 3, respectively (Figures 11e–l).

## 4 Discussion

This study systematically evaluated the effects of 13.5 days of in-orbit flight aboard SJ19 on the physiological characteristics, growth kinetics, stress tolerance, cell phenotypes, and intra- and extracellular enzymatic activities of *BL* strains, and investigated its stability during 12 months of storage at −80 °C after returning to the ground. The experiment set up three groups: FS, carried in orbit for 13.5 days by SJ19, was packaged in single-dose sachets due to satellite constraints. GS1 received identical packaging, whereas GS2 remained in unopened commercial bottles. Comparisons between GS1 and GS2 revealed the effects of single-dose packaging, while FS versus GS1 distinguished the specific biological impacts of spaceflight.

The study found that spaceflight was associated with inhibitory effect on the growth, proliferation and partial metabolic functions of the strains, with some inhibitory effects showing dynamic recovery as storage time increased. In contrast, spaceflight was associated with significantly enhanced biofilm formation and adhesion capacities of the strain, and these increases persisted and were further amplified with prolonged storage relative to baseline. Two-way ANOVA confirmed prominent interactive effects between treatment group and storage duration on bacterial physiological properties. Variance analysis showed that storage duration dominated the phenotypic variation of nearly all indicators, excluding *β*-AL activity.

At 0 months post-recovery, the MCD of the FS group was comparable to that of the GS1 group. All three groups decreased significantly at 6 months of storage, but increased notably at 12 months, returning to levels similar to those at 0 month. The FS and GS1 groups remained comparable throughout, indicating that spaceflight did not cause persistent impairment to the initial growth capacity of the strain.

The lag phase represents the adaptive period during which bacteria remodel their physiology to support subsequent rapid proliferation (Fernández-Martínez et al., 2024), and prolonged lag time is a typical indicator of sublethal microbial injury (Smelt et al., 2002). In this study, the FS group exhibited significantly extended lag phases at 0- and 6-months post-flight compared with the GS1 group, suggesting that spaceflight induced physiological damage in *BL*. However, the lag time of all groups shortened markedly at 12 months, and the difference between FS and GS1 diminished, indicating gradual recovery over storage (Arroyo-Moreno et al., 2025). The μ_max_ decreased progressively across storage time, with no significant difference between FS and GS1 throughout the entire storage period. LCC of the FS group were consistently lower than those of GS1, with significant reductions observed at 6 and 12 months. Along with prolonged storage, viable counts gradually recovered in all groups; specifically, GS1 showed a significant increase, whereas FS recovered significantly only at 12 months. Consistent with these kinetic alterations, spaceflight imposed a plastic and non-permanent impact on bacterial growth. Following relief from space stress and prolonged low-temperature storage, the intrinsic growth capacity of the damaged strains was progressively restored. Notably, spaceflight-induced microbial responses are highly variable, as previous studies have reported enhanced growth performance in space-cultured *Escherichia coli*, including shorter lag phases, longer exponential growth, and higher MCD relative to ground controls (Klaus et al., 1997).

At present, no spaceflight-related reports directly comparable to the long-term stability of the strains in this study after returning to the ground have been retrieved, but the NASA Twin Study records may have certain reference value. The results showed that after nearly 1 year of spaceflight, the intestinal microbiota composition of one of the identical twin astronauts changed significantly. Longitudinal analysis showed that most of the changed biological indicators (including microbiota composition) gradually recovered to near the pre-flight baseline level within about 6 months after returning to the ground. This findings demonstrated that multiple human physiological systems possess adaptive resilience and recovery potential under long-duration spaceflight stress (Garrett-Bakelman et al., 2019).

The growth inhibition observed in this study is consistent with the research results of similar Gram-positive bacteria. For example, the growth rate of *Bacillus subtilis* TD7 was significantly slowed down after spaceflight (Qin et al., 2025); the survival number of *Staphylococcus epidermidis* cultured on the International Space Station (ISS) was also significantly lower than that of the ground control group (Fajardo-Cavazos and Nicholson, 2016). In contrast, Gram-negative bacteria often show stronger adaptability and proliferation ability in the space environment. For example, the cell count of *Escherichia coli* cultured on the ISS was 13 times higher than that of the ground control (Kacena et al., 1999); compared with the normal gravity control group, the final cell density of *Pseudomonas aeruginosa* grown during spaceflight increased when the phosphate concentration in the medium was low and the oxygen supply was reduced (Kim et al., 2013).

In addition to the inhibition of growth and proliferation, spaceflight also had a significant and persistent inhibitory effect on the *α*-AL activity of the strains. FS exhibited consistently lower *α*-AL activity than GS1 at 0, 6, and 12 months post-recovery, and all groups showed markedly reduced enzyme activity at 6 and 12 months compared with 0 month. Consistent with the results of this study, the lipopeptide production of *Bacillus subtilis* TD7 also significantly decreased after spaceflight (Qin et al., 2025). In contrast, existing studies have shown that spaceflight can significantly increase the types and activities of secondary metabolites of *Beauveria bassiana* (Zhang et al., 2022). As an important type of hydrolase, *α*-AL has wide application value in fields such as plant physiology, food industry, and wine brewing. Its reduced activity may reflect the reprogramming or decreased efficiency of energy metabolism pathways caused by spaceflight (Domnin et al., 2022).

After distinguishing intracellular and extracellular *α*-AL activities, we found that at 0-month post-recovery, FS showed significantly lower intracellular *α*-AL activity but remarkably higher extracellular *α*-AL activity than GS1. In *BL*, *α*-AL is a typical secreted protein, and most active enzyme should theoretically be present in the supernatant, with only a small amount retained intracellularly during synthesis or translocation (Strach et al., 2023). However, intracellular *α*-AL activity was 2.6-fold higher than extracellular activity in GS2, and an even higher ratio was observed in GS1 and FS, which may stem from inherent differences in secretion efficiency or methodological bias. The distinct intracellular–extracellular distribution pattern in FS—low intracellular but high extracellular *α*-AL activity—may be attributed to secretion stress responses and increased cell membrane permeability induced by spaceflight stress (Sun et al., 2024; Yasin et al., 2024).

At 6 months, both intracellular and extracellular *α*-AL and *β*-AL activities in FS remained significantly lower than in GS1. By 12 months, no significant differences were observed between the two groups. Together with growth characteristics, these results indicate that spaceflight likely caused sublethal injury in *BL*.

The above-mentioned metabolic inhibition and growth slowdown may be related, together forming the physiological adaptation strategy of *BL* to cope with space stress, that is, prioritizing the allocation of resources to survival and colonization rather than rapid proliferation. A similar study showed that *Bacillus subtilis* TD7 had slowed growth rate and decreased lipopeptide production after spaceflight, suggesting that it adapts to the stress environment by reallocating metabolic resources, prioritizing maintaining survival rather than rapid proliferation (Qin et al., 2025). In addition, Zhan et al. (2023) confirmed that under 2-phenylethanol toxicity stress, *BL* also preferentially activates stress resistance, repair and homeostasis maintenance systems rather than rapid proliferation, indicating that the bacterium adopts a “survival-first” adaptation strategy in stressful environments (Zhan et al., 2023).

In addition, in the early stage after returning to the ground, both the growth rate and *α*-AL activity of the GS1 group showed an increasing trend compared with the GS2 group. This background can clearly attribute the growth inhibition and *α*-AL activity reduction of the FS group compared with the GS1 group to the specific effect of the spaceflight environment, rather than the impact of packaging treatment.

In sharp contrast to growth inhibition, spaceflight significantly and persistently enhanced the biofilm formation and adhesion ability of *BL* relative to baseline, with distinct stability patterns between the two traits. In the early stage after returning to the ground, both biofilm formation ability and adhesion ability were significantly higher in the FS group than in the GS1 group. Such enhancing effects remained stable after 6 and 12 months of storage at −80 °C, although the magnitude of the increase in biofilm formation gradually diminished over time. At 6 months, FS still exhibited significantly greater biofilm formation and adhesion compared with GS1. By 12 months, the difference in biofilm formation between FS and GS1 was no longer statistically significant, whereas adhesion in the FS group remained significantly higher than in the GS1 group. This suggests that the biofilm enhancement phenotype induced by the space environment has a certain “memory effect,” but this memory fades over time. Previous studies have shown that spaceflight can significantly affect the biofilm formation ability of microorganisms, but its effect is not single but has obvious strain specificity. For example, long-term spaceflight can significantly enhance the biofilm formation ability of *Staphylococcus warneri* (Bai et al., 2019); on the contrary, the biofilm formation ability of *Acinetobacter baumannii* and *Acinetobacter schindleri* was significantly reduced after spaceflight (Zhao et al., 2019; Bai et al., 2022). However, an important finding of this study is that the space-induced biofilm enhancement phenotype gradually attenuates with the extension of storage time, showing a decline in the “memory effect.” This phenomenon has rarely been reported in detail in previous literatures, providing a new perspective for understanding the phenotypic stability induced by the space environment. Existing space microbiology studies mostly focus on phenotypic observation immediately after flight or in a short period of time, and there are few longitudinal tracking data, which makes it difficult to directly compare and analyze with the results of this study.

The study found that the enhanced biofilm formation ability induced by spaceflight has dynamic change characteristics, suggesting that the spaceflight-induced biofilm enhancement may mainly come from epigenetic modification or transient regulation of gene expression, rather than stable genetic mutations. This inference is supported by some literatures. For example, Morrison et al. (2019) reported that the expression of biofilm formation-related genes (such as *srfAA-AB-AC-AD-ycxA* and *tapA-sipW-tasA* operons) of *Bacillus subtilis* was significantly upregulated in the ISS environment. This proves that the space environment can directly regulate the gene network related to biofilm synthesis. Although this study did not detect the final phenotype, this molecular evidence suggests that the space environment affects the potential characteristics of microorganisms by triggering specific gene expression programs, and this regulation is usually flexible and dependent on environmental signals, which lends support to our inference that phenotypic changes may be mediated by epigenetic or transcriptional regulation (Morrison et al., 2019). In addition, studies on the adaptability of microorganisms to stress factors also point out that phenotypic changes may be a plastic adaptive response, and when the inducing stress disappears, the phenotype may retract (Rapacka-Zdonczyk, 2026; Tan et al., 2022; Burns et al., 2021).

In contrast, the enhancement of adhesion ability in the FS group showed higher stability. At 0, 6, and 12 months, its adhesion ability was 58.84, 54.30, and 45.97% higher than that of the GS1 group, respectively (all *p* < 0.0001). Although the increase amplitude slightly decreased, it remained at a high level overall. This may suggest that the space environment induced more persistent changes in the strains, even possibly gene mutations, which is expected to help probiotics reside in the intestinal tract and exert probiotic effects. Multiple studies have confirmed that the adhesion characteristics of probiotics can effectively extend their residence time in the gastrointestinal tract, giving the strains more opportunities to interact with host intestinal epithelial cells, immune cells, and intestinal microbial communities to more persistently regulate host immune responses, enhance intestinal barrier function, produce beneficial metabolites (such as short-chain fatty acids), and competitively repel the invasion of pathogenic microorganisms through the “occupation effect” to exert probiotic effects (Singh et al., 2022; Moroz et al., 2021).

It is worth noting that the adhesion ability of the GS1 group was significantly lower than that of the GS2 group at all time points, indicating that the single-dose packaging treatment itself may weaken the adhesion potential of the strains. In this context, the adhesion ability of the FS group was still much higher than that of the GS1 group, which further highlights the specific enhancing effect of spaceflight on the adhesion ability of the strains.

In our previous study, we conducted a spaceflight experiment using the Shenzhou-15 spacecraft to expose *BL* to the orbital environment for a duration of 6 months. The results of changes in strain physiological activity showed that its growth activity was significantly weakened after spaceflight, which was consistent with the trend of the results of this study; however, in terms of biofilm formation ability, cell adhesion ability, and *α*-AL activity, the change trends after spaceflight were opposite to those of this study (Li et al., 2026). These discrepancies are likely attributed to variations in flight conditions, particularly regarding key environmental parameters such as microgravity, space radiation, and the duration of orbital exposure. The Shenzhou-15 manned spacecraft employed an active temperature control and environmental regulation system for biological samples. During its 186-day orbital stay, the microgravity level was maintained better than 1 × 10^−6^ g, and the crewed cabin provided substantial radiation shielding, keeping the annual radiation dose below 50 rad (Yu, 2023). In contrast, the SJ19 satellite utilized a passive, non-powered loading mode. Its orbital mission lasted 13.5 days with a microgravity level of 1 × 10^−7^ g, and the measured space radiation dose ranged from approximately 0.148 rad (Si) to 0.300 rad (Si).

Previous studies have shown that long-term low-temperature storage may have an imperceptible impact on the physiological state of strains (Alabdullatif, 2024; Chang et al., 2025). Therefore, the attenuation of biofilm formation ability in this study may also be related to the frozen storage process.

This study presents the first investigation into the dynamic changes in the physiological activity of *BL* over a 12-month post-flight storage period on the ground, providing experimental evidence for evaluating the long-term stability of space-mutated strains. However, certain limitations in this study should be acknowledged. First, the lack of multi-omics data precluded a systematic elucidation of the molecular mechanisms underlying space environment-mediated regulation of physiological activity at the transcriptional, translational, and metabolic levels. Second, the intrinsic mechanisms governing the physiological alterations induced by the SJ19 and the subsequent stability changes during 12 months of ground storage were inferred based on existing literature, rather than through direct mechanistic validation. Additionally, although two ground control groups were established, the absence of multiple replicate flight experiments makes it difficult to definitively isolate the independent contribution of the space environment to the observed physiological changes. Future studies should integrate multi-omics approaches—including transcriptomics, proteomics, and metabolomics—to systematically dissect the molecular regulatory mechanisms driving phenotypic variations. Furthermore, *in vivo* animal models should be employed to verify the intestinal colonization ability, probiotic efficacy, and biosafety of *BL* following spaceflight, thereby providing a more comprehensive theoretical and data foundation for the future development and application of space-mutated probiotics.

This study systematically analyzed the effects of 13.5 days of on-orbit flight via the SJ19 satellite on the growth characteristics, stress resistance, cell surface properties, and extracellular/intracellular enzyme activities of the *BL* strain, as well as its stability during 12-month storage at −80 °C after return to Earth. The results indicated that spaceflight was associated with altered growth metabolism and surface properties of the *BL* strain, inducing relative growth retardation, decreased viable cell counts and inhibited *α*-AL activity, together with enhanced bacterial biofilm formation and cell adhesion. During the 12-month storage period, the space-exposed strain exhibited time-dependent recovery in growth performance, continuous reduction in *α*-AL activity, and upregulation of biofilm formation and adhesion abilities relative to baseline. These findings suggest that the impact of the space environment on the strain is plastic and reversible, providing new empirical data for understanding the space adaptation mechanism of microorganisms, and also laying an important preliminary research foundation for the future development of stable and efficient space medical probiotic preparations suitable for long-term space missions.

## Data Availability

The original contributions presented in the study are included in the article/supplementary material, further inquiries can be directed to the corresponding authors.
